# Heterojunction FeTiO_3_/BiOCl Photocatalytic Polymer Film in an Airlift Reactor: Efficient Visible-Light Degradation of Pharmaceutical Pollutant

**DOI:** 10.3390/polym18101246

**Published:** 2026-05-20

**Authors:** Nergiz Kanmaz, Nese Cakir Yigit, Özlem Tuna

**Affiliations:** 1 Department of Chemical Engineering, Faculty of Engineering, Yalova University, 77200 Yalova, Turkey; nzeynep.kanmaz@yalova.edu.tr; 2 Department of Polymer Materials Engineering, Faculty of Engineering, Yalova University, 77200 Yalova, Turkey

**Keywords:** FeTiO_3_, BiOCl, PVDF, polymeric thin film, photocatalysis

## Abstract

The development of durable and practical polymer-supported photocatalytic materials that are suitable for use in continuous-flow systems has become an increasingly pressing issue in the field of water treatment. In this study, FeTiO_3_/BiOCl heterojunction structures were synthesized at different ratios and integrated into a poly(vinylidene fluoride) (PVDF) matrix to develop photocatalytic thin-film systems. The resulting materials were characterized by Fourier-transform infrared spectroscopy (FTIR), X-ray diffraction (XRD), scanning electron microscopy (SEM), thermogravimetric analysis (TGA), and UV–visible diffuse reflectance spectroscopy (UV-DRS) analyses. In photocatalytic experiments conducted under visible light, a 66.3% removal of doxycycline was achieved for pristine FeTiO_3_ within 180 min, whilst the FTO@BiOCl(III) composite reached 74.4%. In the PVDF-based thin-film system, the film catalyst demonstrated a removal efficiency of 68.9%. When the pH effect was investigated, the highest total removal of 90.3% was achieved under pH 6.0 conditions. Radical scavenging experiments revealed that superoxide radicals were the predominant active species (a decrease to 30.5% in the presence of benzoquinone (BQ). In experiments conducted in the air-lift reactor system, the P-FTO@BiOCl(III) film achieved approximately 65% removal after 9 h and maintained its structural stability. The PVDF-supported FeTiO_3_/BiOCl heterojunction thin-film system offers a noteworthy alternative for environmental applications due to its suitability for continuous systems, structural stability and effective photocatalytic performance.

## 1. Introduction

Recently, ferrite titanate (FeTiO_3_) has attracted the interest of researchers in various photocatalytic wastewater treatment applications, particularly the degradation of antibiotics [1]. Since ilmenite-based perovskite can effectively absorb visible light with a bandgap value of approximately 2.3 eV [2], it exhibits outstanding properties: namely, low cost and chemical stability. However, the practical photocatalytic efficiency of FeTiO_3_ is struggling with some drawbacks: the high rate of photo-induced charge carrier recombination and poor charge transfer kinetics cause the generated electron–hole pairs to be eliminated before they can form reactive oxygen species (ROS), which in turn significantly reduces the quantum yield and active area utilization [3]. To overcome these limitations, the most effective strategy is to form a heterojunction with a second semiconductor possessing a complementary band structure [4]. While several semiconductor candidates have been explored for pairing with iron-based photocatalysts, including g-C_3_N_4_-, TiO_2_-, and Fe_2_O_3_-based systems, these materials often suffer from narrow visible light absorption, rapid charge recombination, or limited chemical stability under prolonged irradiation [5]. In this context, bismuth oxychloride (BiOCl) emerges as a particularly advantageous partner. BiOCl has been demonstrated to outperform conventional photocatalysts; Zhang et al. reported that BiOCl exhibited superior photocatalytic degradation activity compared to TiO_2_, and subsequent studies confirmed that BiOCl nanoplates showed 1.7 times higher activity than TiO_2_ toward organic pollutant degradation [6]. This superior performance stems from the strong internal electric field created by its tetragonal layered [Bi_2_O_2_]^2+^ and [Cl^−^] structure, which accelerates the separation of photo-excited charge carriers, as well as its contribution to the adsorption capacity via surface oxygen vacancies and functional group richness [7,8,9]. Although the relatively large bandgap of BiOCl (~3.2 eV) limits its standalone activity under visible light, constructing heterojunctions with narrow-bandgap semiconductors such as FeTiO_3_ is a well-established strategy to extend light absorption into the visible range while preserving its inherent advantages for charge separation [6]. Indeed, the approach of enhancing perovskite-structured compounds by forming heterojunctions with BiOCl is attracting increasing interest in the literature; for example, the synthesis of the Bi_5_Ti_3_FeO_15_/BiOCl nanocomposite has demonstrated that the synergy between oxygen vacancies and the heterojunction significantly enhances catalytic removal of organic pollutants [10].

On the other hand, polymeric thin films have emerged as highly versatile platforms for immobilizing photocatalytically active inorganic materials, offering critical practical advantages over conventional powder-based systems, including ease of recovery, reusability, scalability, and the elimination of post-treatment separation steps [11,12]. Furthermore, the heterojunction architecture, including polymer thin films, promotes efficient charge separation at the interface between two semiconductors with staggered band alignments, thereby suppressing electron–hole recombination and extending the lifetimes of photogenerated charge carriers under visible light irradiation, ultimately enabling continuous and stable operation under realistic wastewater treatment conditions [13,14,15]. At this point, polyvinylidene fluoride (PVDF) has been presented as a highly suitable polymer structure due to its chemical resistance, thermal stability, mechanical strength, and ability to form thin films. Unlike other membrane-forming polymers such as polyethersulfone (PES) or polysulfone (PSf), which serve primarily as passive support matrices, PVDF is the most utilized polymer in wastewater treatment membranes, due to its excellent properties including its hydrophobic nature, antifouling properties, aging resistance, mechanical strength, high thermal resistance, and mechanical and chemical stability, while simultaneously functioning as an active component through its intrinsic piezoelectric response. These characteristics are particularly advantageous for the phase-inversion method [16,17,18]. Critically, the electric field produced in inorganic fillers in the PVDF matrix is used to improve the separation of the photoinduced carriers of metal oxide nanostructures, and the piezoelectric field generated in PVDF by mechanical action can improve the catalytic efficiency of photogenerated carriers, establishing a direct structure–property relationship between the polymer phase composition and the photocatalytic performance of the composite film [19]. The electroactive β-phase of PVDF produces an internal electric field that promotes charge separation under light irradiation and enhances photocatalytic degradation [20]. In this regard, interest in PVDF-based composites has increased due to their inherent β-phase, which enhances charge segregation when combined with photocatalytic semiconducting materials [21]. Importantly, the incorporation of inorganic fillers into the PVDF matrix during the phase-inversion process has been shown to promote the transition from the non-polar α-phase to the electroactive β-phase, further amplifying the piezoelectric contribution to charge separation and directly influencing photocatalytic activity [22]. Recent research has demonstrated that PVDF-based photocatalytic membranes are effective in removing antibiotics from water [23]. For example, g-C_3_N_4_/β-PVDF membranes can degrade tetracycline (TC), achieving removal rates greater than 93% [24]. Incorporation of N-CQDs/Bi_2_WO_6_ into PVDF-HFP membranes further enhances antibiotic removal by utilizing both heterojunction and piezoelectric effects for efficient charge separation [25]. Furthermore, TiO_2_/PVDF films prepared via phase inversion with 6% TiO_2_ in a 12.5% PVDF blend show optimal performance under specific conditions, such as pH 5, and exhibit high TC degradation efficiency [26]. Overall, these results highlight the considerable potential of PVDF-based composite membranes as efficient, reusable, and scalable photocatalysts for remediating antibiotic-contaminated water [27].

As a promising platform for polymer-based heterostructure samples in thin-film form, air-lift reactors have gained considerable attention due to their unique hydrodynamic properties and energy-efficient operation in long-term removal processes [28]. This design provides several advantages for visible-light-driven thin film: (i) it decreases mechanical stress on the active film, enabling stability of the film during photocatalysis, (ii) it facilitates mass transfer between the pollutant and the photocatalytic surface, and (iii) the air used as a mixing agent in the reactor system improves light penetration and oxygen availability [28,29,30,31]. In this context, the air-induced mechanical agitation in the airlift reactor is particularly relevant for PVDF-based composite films, as the fluid-induced mechanical stimulation is expected to activate the piezoelectric response of the β-phase PVDF matrix, generating an additional internal electric field that synergistically enhances charge carrier separation alongside the FeTiO_3_/BiOCl heterojunction—a unique functional advantage that is specific to the piezoelectric polymer matrix and cannot be replicated by passive support materials [32]. According to these, PVDF-based membranes incorporating FeTiO_3_/BiOCl heterojunction into an air-lift reactor system could represent as a novel and practical approach for efficient photocatalytic degradation under visible light irradiation, combining the structural stability and flexibility of the film with the superior mixing, increased active surface area, and improved oxygen mobility.

Despite the growing interest in PVDF-based photocatalytic composite films and heterojunction systems for antibiotic removal, several critical gaps remain in the literature. While numerous studies have explored PVDF-based membranes incorporating single-component photocatalysts such as TiO_2_ [26] or g-C_3_N_4_ [24], the integration of dual-semiconductor heterojunctions—particularly FeTiO_3_/BiOCl—into piezoelectric PVDF films remains largely unexplored. Furthermore, although doxycycline (DOX) is among the most widely detected and environmentally persistent antibiotics in wastewater [33], its photocatalytic degradation using immobilized heterojunction-based polymer films has received considerably less attention compared to tetracycline.

In this study, FeTiO_3_ perovskite catalyst was incorporated with BiOCl particles at varying concentrations via a facile mixing process, followed by surface modification. Then, the FeTiO_3_@BiOCl was incorporated into PVDF-based thin film via the phase inversion method. The structural, optical, and morphological characteristics of the hybrid materials in powder and thin film were determined by FTIR, TGA, XRD, SEM, and UV-DRS analyses. The photocatalytic performance of the catalysts was evaluated by degrading doxycycline (DOX) under visible light irradiation: powder catalysts were tested in a batch system, whereas film samples were assessed in an air-lift reactor. The effects of BiOCl amount and solution pH on their catalytic efficiency were examined in the antibiotic decomposition. Furthermore, the degradation mechanisms were proposed by evaluating studies on trapping agents. Finally, the PVDF- FeTiO_3_@BiOCl catalytic system was tested for the degradation of doxycycline in an air-lift reactor system, and the reusability of the catalyst was assessed through comprehensive characterization of the as-used thin film (FTIR, XRD, and SEM). This study introduces a durable and innovative polymeric thin film with strong potential for continuous and practical water treatment applications.

## 2. Materials and Methods

### 2.1. Materials

Monoethylene glycol (MEG), bismuth nitrate penta hydrate (Bi(NO_3_)_3_.5H_2_O), potassium chloride (KCl), iron nitrate nona hydrate (Fe(NO_3_)_3_.9H_2_O), titanium n-butoxide, glacial acetic acid, sodium hydroxide (NaOH), hydrochloric acid (HCl-37%), ethylenediaminetetraacetic acid disodium (EDTA-2Na), benzoquinone (BQ) and isopropyl alcohol (IPA) and absolute ethanol were purchased from Merck KGaA (Darmstadt, Germany). Doxycycline hyclate was obtained from Sigma-Aldrich (St. Louis, MO, USA). Polyvinylidene fluoride (PVDF) was purchased from Sigma-Aldrich (St. Louis, MO, USA) ((–CH_2_CF_2_-)_n_, average M_w_ ~275,000, average M_n_ ~107,000) in the form of pellets. N,N-dimethylformamide (DMF, ≥99.8%, Sigma-Aldrich (St. Louis, MO, USA)) was used without further purification.

### 2.2. Synthesis of Photocatalysts

BiOCl was synthesized using a controlled precipitation method: 3 mmol of bismuth nitrate pentahydrate was stirred at room temperature in 25 mL of monoethylene glycol (MEG) until a homogeneous solution was obtained. Simultaneously, a solution of 3 mmol potassium chloride in 25 mL of distilled water was prepared in a separate vessel. The molar ratio between bismuth nitrate pentahydrate and potassium chloride was fixed at 1:1, according to the stoichiometric requirement for BiOCl formation. Subsequently, the KCl solution was added dropwise at a constant rate to the MEG-based bismuth precursor solution, and the mixture was stirred continuously at 70 °C for 2 h to ensure controlled crystallization. After the reaction period, the resulting white precipitate was separated by filtration and subsequently washed successively with MEG and distilled water to remove unreacted components. Finally, the obtained BiOCl was dried in an oven at 80 °C.

FeTiO_3_ perovskite was synthesized using a controlled sol–gel method. In the experimental procedure, 26.57 g (65.8 mmol) of iron(III) nitrate nonahydrate was dissolved in 20 mL of distilled water to prepare a homogeneous solution. The 28 mL (82.8 mmol) of titanium n-butoxide used as the titanium source was diluted in 40 mL of ethanol, and 10 mL of glacial acetic acid was slowly added to control the hydrolysis rate. That is, the molar ratio between iron(III) nitrate nonahydrate and titanium n-butoxide was adjusted to approximately 1:1.25 to compensate for precursor loss during hydrolysis and to ensure the stoichiometric formation of FeTiO_3_ perovskite. With the addition of this modifying agent, a ligand exchange reaction (Equation (1)) was carried out, and the mixture was stirred for 30 min.

Ti-OR + CH_3_COOH ⟶ Ti-O-Ac + ROH (1)

Subsequently, the prepared iron nitrate solution was added dropwise to the titanium complex, and the mixture was stirred continuously for 1 h to ensure molecular homogeneity. The resulting powders were heated to 700 °C at a heating rate of 5 °C/min to facilitate crystallization and were calcined at this temperature for 6 h to obtain FeTiO_3_ powders.

FeTiO_3_/BiOCl (FTO@BiOCl) heterojunction composites were prepared using a mixing method based on surface modification at three different mass ratios. During the synthesis process, in the first stage, 15 mL of ethyl alcohol and 1 mL of 1 M NaOH were added to the specified amounts of FeTiO_3_ powder to enhance surface activity; the mixture was then subjected to ultrasonication for 10 min to obtain a homogeneous suspension and prevent particle aggregation. Subsequently, BiOCl powder in the relevant proportions was incorporated into the system, and the mixture was sonicated for an additional 10 min; to maximize the physical interactions between the components, the mixture was maintained at 50 °C under continuous stirring for 1 h. The hybrid structures obtained at the end of the process were dried at 70 °C after solvent removal. The samples were labeled FTO@BiOCl(I), FTO@BiOCl(II) and FTO@BiOCl(III) according to the mass ratios of FTO:BiOCl of 0.5:0.5, 0.7:0.3 and 0.3:0.7 g. The schematic representation of BiOCl, FeTiO_3,_ and FeTiO_3_@BiOCl heterostructures was summarized in Figure S1.

### 2.3. Synthesis of Polymer-Based Active Thin Films

**Figure 1:**
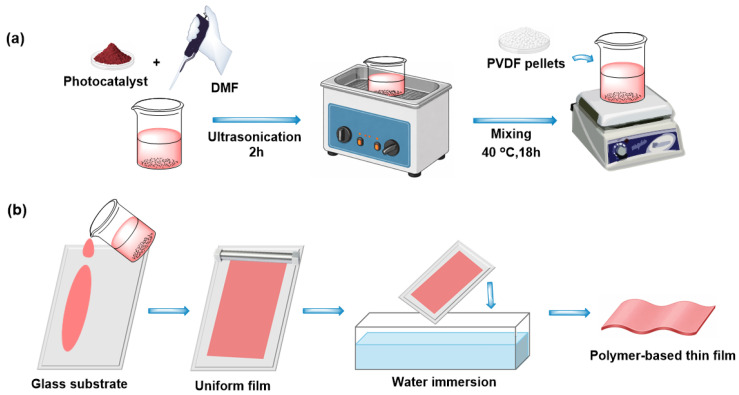
Schematic representation of the preparation steps of polymer-based active thin films from solution (**a**) to film (**b**).

The polymer-based thin films were prepared by the phase-inversion method. For this purpose, N,N-dimethylformamide (DMF) was chosen as the solvent due to its strong solvating ability toward polyvinylidene fluoride (PVDF) and its compatibility with various powder components, enabling homogeneous polymer dissolution and uniform film formation. BiOCl, FeTiO_3_, and BiOCl/FeTiO_3_ heterostructures (30, 50, and 70 wt% BiOCl) were employed as photocatalysts. In this procedure, as a first step, 0.1 g of photocatalyst was dissolved in 4 mL of DMF, and the mixture was ultrasonicated for 2 h to disperse the powder homogeneously in the solvent. As a second step, 0.20 g of PVDF was added to the medium, and the solution was stirred magnetically for 18 h at 40 °C to ensure complete dissolution of PVDF (Figure 1a). At the final step, a 100 μm-thick film was then cast onto a glass substrate using an applicator, followed by immersion in a water bath to remove the film from the glass surface. The polymer film was laid on the smooth surface using binder clips and dried in the fume hood for 24 h (Figure 1b). The same procedure was used to prepare other film formations with different photocatalyst types. The obtained thin films are coded as P-BiOCl, P-FTO, P-FTO@BiOCl(I), P-FTO@BiOCl(II), and P-FTO@BiOCl(III), respectively, as defined above for the photocatalyst used for film formation.

### 2.4. Material Characterization

The structural, morphological and optical properties of the prepared BiOCl, FeTiO_3_, FTO@BiOCl and P-FTO@BiOCl composites were investigated using various advanced analytical techniques. The crystal structures and phase compositions of the materials were determined in the 5° to 90° (2θ) range using an X-ray diffractometer (XRD, Rigaku SmartLab) operating at 40 kV and 30 mA with Cu-Kα radiation, with a step size of 0.02° and a scan rate of 2°/min. Fourier transform infrared spectroscopy (FT-IR, Perkin Elmer Spectrum 100) analyses were performed in the 400–4000 cm^−1^ wavelength range at a spectral resolution of 4 cm^−1^ with 32 accumulated scans to identify functional groups and elucidate chemical bond structures. The thermal stability and mass loss behavior of the synthesized catalysts were monitored by thermogravimetric analysis (TGA, Mettler Toledo TGA/SDTA 851) under a nitrogen atmosphere at a heating rate of 10 °C/min from 25 °C to 900 °C with a nitrogen flow rate of 50 mL/min and a sample mass of approximately 5 mg. The surface morphology of the nanocomposites was analyzed using high-resolution images obtained via scanning electron microscopy (SEM, JEOL 6610), after sputter-coating the samples with a thin gold (Au) layer (~10 nm) to prevent charging, at an accelerating voltage of 15 kV. The elemental composition and spatial distribution of the constituent elements were determined by energy-dispersive X-ray spectroscopy (EDS) using an Oxford Instruments X-MaxN 50 mm^2^ detector integrated with the SEM system, operated at the same accelerating voltage. UV–Vis diffuse reflectance spectroscopy (UV-DRS, SHIMADZU UV-2600i) measurements were carried out to investigate the light absorption behavior in the UV–visible region. The as-obtained reflectance data were transformed into absorption spectra using the Kubelka–Munk function, enabling the estimation of optical band gap energies. In addition, photoluminescence (PL, Edinburgh Instruments FLS1000 spectrometer) analysis was conducted to assess the separation and recombination behavior of photogenerated electron–hole pairs, which plays a critical role in photocatalytic efficiency. The measurements were performed at room temperature with an excitation wavelength of 325 nm, and the emission spectra were collected in the range of 350–700 nm with a spectral resolution of 1 nm.

### 2.5. Photocatalytic Experimental Studies

**Figure 2:**
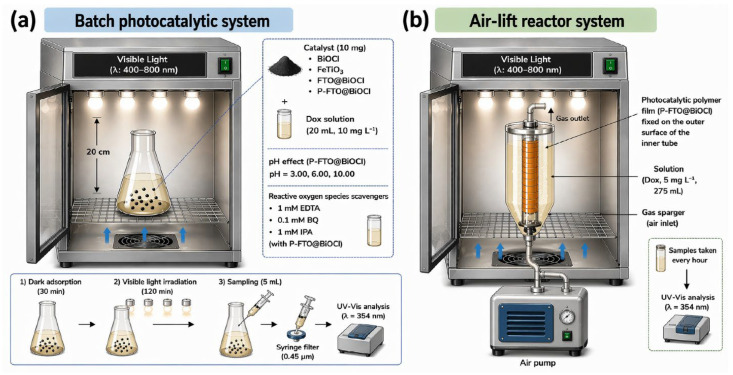
Schematic representation of photocatalytic experimental set−up as (**a**) batch system and (**b**) air-lift reactor system. Blue arrows indicate the direction of airflow (ventilation/cooling) within the irradiation chamber.

A cabinet with four visible lamps (Osram 150 W, λ: 400–800 nm) was used for the photocatalytic DOX removal experiments and the flask was positioned 20 cm away from the lamps in the chamber. To prevent an increasing ambient temperature, two cooling fans positioned at the bottom of the cabinet were applied to cool the system (Figure 2a). After weighing 10 mg of BiOCl, FeTiO_3_, FTO@BiOCl, and P-FTO@BiOCl composites, 20 mL of 10 mg L^−1^ DOX was added. In pH effect experiments, 20 mL was added to 10 mg of P-FTO@BiOCl (1 mm × 2 mm) after the pH of DOX solutions was changed to 3.00, 6.00, and 10.00. The 10 mg L^−1^ doxycycline solutions were made by adding 1 mM ethylenediaminetetraacetic acid (EDTA), 0.1 mM benzoquinone (BQ), and 1 mM isopropyl alcohol (IPA) in order to investigate the effects of reactive oxygen species. Then, 10 mg of P-FTO@BiOCl was mixed with 20 mL of a 10 mg L^−1^ DOX solution. To achieve adsorption/desorption equilibrium, the samples were first agitated for 30 min in the dark system. After turning on the visible light, a 5 mL sample was collected after 120 min and passed through a syringe filter with a pore diameter of 0.45 mm. The filtrate’s DOX concentration in the filtrate was determined at 354 nm using a UV–Vis spectrophotometer.

A specially designed airlift photoreactor set-up (Figure 2b) was employed to evaluate the practical application potential and performance of the prepared heterojunction hybrid polymeric photocatalytic films in a continuous system. In the experimental setup, the flexible polymeric photocatalytic film (10 × 20 cm) was secured to the outer surface of the inner tube located at the center of the reactor to maximize light interaction. Then, 275 mL of a DOX solution at a concentration of 5 mg L^−1^ was added to the reactor as a model contaminant. Continuous air flow, provided by an air pump integrated into the lower part of the system, ensured the regular circulation of the solution within the reactor. By maintaining a constant circulation rate, both the system’s hydrodynamic stability and the degradation performance of the proposed polymeric photocatalyst under dynamic conditions were investigated. During the photocatalytic reaction, samples were taken from the reactor at specified time intervals (every hour), and changes in contaminant concentration were monitored using spectrophotometric methods.

## 3. Results and Discussion

### 3.1. Characterization

**Figure 3:**
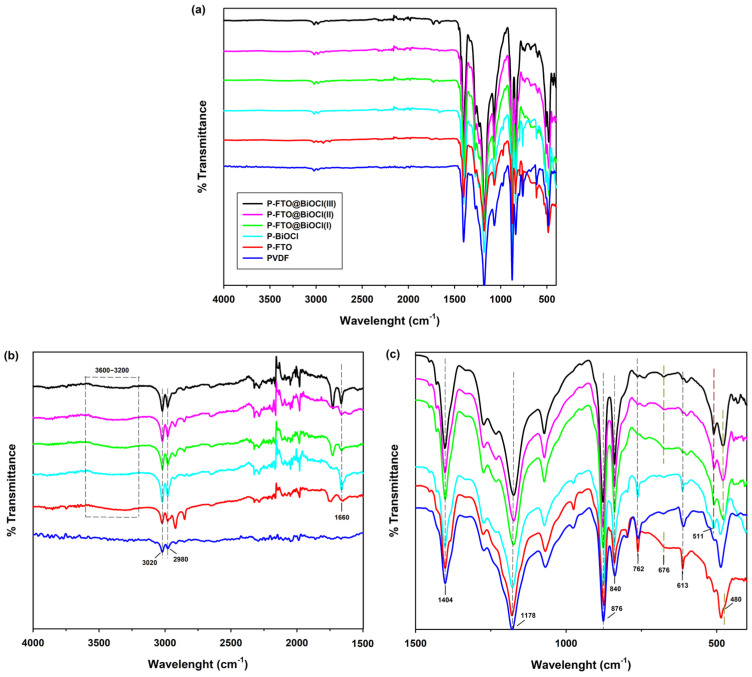
FTIR spectra of polymer−based thin films. (**a**) Full spectra. (**b**,**c**) Enlarged spectra.

**Figure 4:**
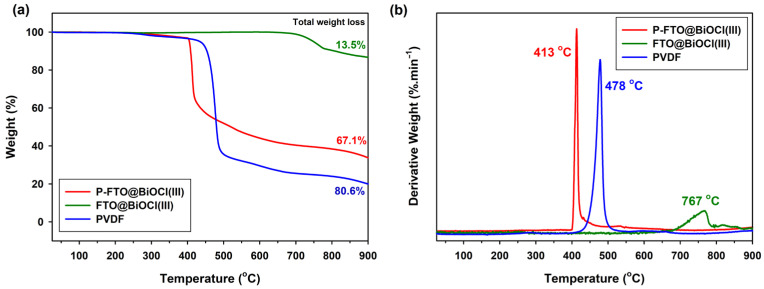
(**a**) TGA thermograms of P-FTO@BiOCl(III), FTO@BiOCl(III), and PVDF and (**b**) DTG thermograms of P-FTO@BiOCl(III), FTO@BiOCl(III), and PVDF.

The chemical composition of polymer-based thin films was thoroughly investigated using FTIR analysis. Figure 3a depicts the full spectrum of polymer-based thin films in the range of 4000–400 cm^−1^. They were enlarged to analyze the peaks deeply, as shown in Figure 3b for the 4000–1500 cm^−1^ region and Figure 3c for the 1500–400 cm^−1^ region. In the enlarged spectrum of the composite thin films, a broad O-H stretching peak was observed at around ~3400 cm^−1,^ which is significantly different from the bare PVDF spectrum (Figure 4b). This marked contrast to the bare PVDF, which showed a significant shift in the spectrum, indicating an increased hydrophilicity that might originate from the inorganic fillers [34]. Additionally, the presence of surface hydroxyl groups originating from fillers was evidenced by the H–O–H bending vibration at ~1660 cm^−1^. The weak bands observed at approximately 3020 and 2980 cm^−1^ were attributed to the asymmetric and symmetric C–H stretching vibrations of the –CH_2_– groups in PVDF, respectively, and were common to both the α and β phases [35]. As depicted in Figure 3c, the other characteristic absorption bands of PVDF were observed at 1404 cm^−1^ (CH_2_ scissoring vibration of α-phase), ~1178 cm^−1^ (symmetric CF_2_ stretching vibration), 876 cm^−1^ (C–C skeletal rocking vibration of β-phase), 840 cm^−1^ (CH_2_ rocking and CF_2_ stretching of β-phase), 762 cm^−1^ and 613 cm^−1^ (CF_2_ bending and skeletal bending of α-phase). In the composite films, the characteristic peaks of PVDF remain dominant, indicating that the incorporation of BiOCl/FeTiO_3_ did not significantly alter the polymer backbone. However, upon incorporation of BiOCl/FeTiO_3_ heterojunctions to the PVDF matrix, a relative intensification of the 840 cm^−1^ band accompanied by an attenuation of the 762 cm^−1^ and 613 cm^−1^ bands was observed, suggesting that the presence of inorganic fillers promotes the transition from the non-polar α phase to the polar β phase within the polymer matrix [36]. Furthermore, additional absorption bands observed in the 400–600 cm^−1^ region could be attributed to the metal–oxygen vibrations of the inorganic components. Specifically, the band at 511 cm^−1^ corresponds to Bi–O stretching in BiOCl [34], while the broad feature was associated with Fe–O at 480 cm^−1^ and Ti–O vibrations at 676 cm^−1^ in FeTiO_3_ [37]. These bands were typically weak due to the lower content of the inorganic phase and partial overlap with PVDF skeletal vibrations. Overall, these FTIR findings reveal two key insights that are critical to the photocatalytic performance of the composite films: the increased hydrophilicity induced by the inorganic fillers facilitates better contact between the catalyst surface and the aqueous reaction medium, while the promoted α-to-β phase transition in PVDF enhances the piezoelectric properties of the polymer matrix, both of which are expected to contribute positively to the overall photocatalytic efficiency.

TGA analysis was performed to evaluate the thermal stability of the synthesized photocatalysts and polymer-based composite films under conditions relevant to photocatalytic applications, where local temperature increases in the reaction medium may occur during prolonged light irradiation. Figure 4 depicts the TGA (a) and corresponding DTG (b) curves of bare PVDF film, BiOCl/FeTiO_3_ heterojunctions in powder form (FTO@BiOCl(III)) and film form (P-FTO@BiOCl(III)) from 25 to 900 °C in nitrogen atmosphere with 10 °C/min heating rate. As shown in Figure 4a, the thermal stability of polymer-based thin films and photocatalysts was evaluated by measuring temperature-dependent weight loss (%). The plot showed the weight loss profiles of each sample across the entire temperature range. The TGA curve of the FTO@BiOCl(III) powder demonstrated excellent thermal stability, a characteristic of fully inorganic composite systems, making it a good candidate for enhancing the thermal stability of polymer matrices [38]. Two stages of weight loss were observed for both bare PVDF and BiOCl/FeTiO_3_ heterojunction containing PVDF (P-FTO@BiOCl(III)) film. As the films were completely dry, no moisture removal was observed, and they remained thermally stable up to around 250 °C. The first weight-loss stage for PVDF and P-FTO@BiOCl(III) films was from 250 °C to 400 °C and approximately 5% weight loss, which was due to the burning decomposition of other volatile residuals. The greatest weight (second-stage) loss occurred between 400 °C and around 550 °C due to the burning degradation of the carbon skeleton [37]. With regard to the incorporation of FTO@BiOCl(III) heterojunctions, Figure 4a indicated that the composite presented lower thermal stability compared to the bare PVDF film, and the onset temperature decreased from 435 °C to 400 °C. Regarding the residual portion, at temperatures between 550 and 900 °C, the P-FTO@BiOCl(III) film exhibited a higher residual production than the bare PVDF film [39]. These measurements indicated that the interactions between PVDF and FTO@BiOCl(III) heterojunctions were consistent and enhanced the thermal stability of the PVDF. As depicted in Figure 4b, the DTG curve of bare PVDF exhibited a strong single-stage degradation peak at 478 °C, consistent with the reported literature [40]. There was a shift in the maximum thermal decomposition peak for the P-FTO@BiOCl(III) film, indicating a decrease in overall crystallinity. The polymer stiffness was decreased with the addition of FTO@BiOCl(III) heterojunction incorporation [23,40,41].

**Figure 5:**
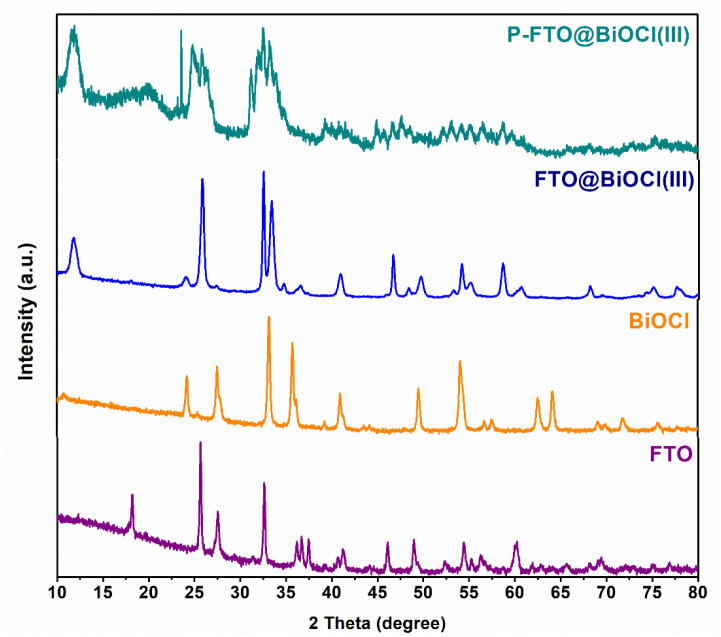
XRD patterns of as-fabricated samples.

The XRD analysis was performed to elucidate the crystalline structures and phase compositions of the as-fabricated FTO, BiOCl, and FTO@BiOCl(III) heterostructures, as well as FTO@BiOCl(III)-included polmeric thin film. As depicted in Figure 5, the relevant pattern of the pristine FeTiO_3_ sample showed that the diffraction peaks located at 2θ ≈ 24°, 33°, 36°, 40°, 49°, 53°, and 61.6° correspond to the (012), (104), (110), (113), (024), (116), and (214) reflection planes, respectively, confirming successfull formation of a rhombohedral crystalline structure (PDF 01-070-6267) [42,43]. On the other hand, minor peaks were observed at approximately 2θ = 17°, 28°and 46°, which were attributed to the formation of Fe^2+^ species, namely Fe_2_Ti_3_O_9_, TiO_2_, and Fe_2_O_3_, respectively [44,45,46]. The formation of the small fractions was due to partial oxidation during perovskite synthesis. In the case of sole BiOCl, characteristic diffraction peaks observed at 2θ = 24° (002), 26° (101), 32° (110), 33° (102), 41° (112), and 47° (200) were well-matched with the tetragonal matlockite-type crystal structure (JCPDS No. 85-0861) [47]. In the FTO@BiOCl(III) composite pattern, the diffraction peaks corresponding to both FeTiO_3_ and BiOCl phases were identified, confirming efficient integration of the two components without the formation of any impurity phases. Furthermore, after the integration of FTO with BiOCl, the specific peak of BiOCl at 11.98° (001), which was assigned to the layered [Bi_2_O_2_]^2+^ structural units, occurred. This could be a sign of internal electric fields generated between the BiOCl-based cationic and the FTO-based anionic layers [48]. Additionally, the XRD pattern of the P-FTO@BiOCl(III) film revealed several significant structural modifications compared to the relevant composite powder: (i) the intensities of the FeTiO_3_ and BiOCl diffraction peaks markedly decreased, which could be due to the encapsulation of inorganic crystallites within the amorphous PVDF matrix, resulting in a partial restriction of long-range crystalline order and (ii) the broadening of diffraction peaks observed at approximately 2θ = 25° and 35°, which was attributed to the partial overlap of the inorganic composite peaks with characteristic reflections of the PVDF polymer matrix. Specifically, the overlapping peak in the ~25° region showed the occurrence of the α-PVDF (120) crystallographic plane at ~25.55° and the (021) reflection at ~26.5–26.6°, both of which were well-established secondary diffraction peaks of the monoclinic α-phase of PVDF [42,43]. Moreover, the peak broadening observed around 35° corresponded to the formation of the α-phase PVDF (200) crystal structure [35,49,50]. The results clearly demonstrated that both the pristine photocatalysts (FeTiO_3_ and BiOCl) and the derived heterostructures, in powder and PVDF-based film forms, were successfully synthesized as designed. In summary, the XRD results provide three key insights into the material design: the emergence of the (001) BiOCl peak upon heterostructure formation suggests the generation of internal electric fields at the FeTiO_3_/BiOCl interface, which is expected to facilitate the separation of photogenerated charge carriers; the absence of impurity peaks in the FTO@BiOCl composite confirms the phase integrity of the heterostructure; and the structural modifications observed in the P-FTO@BiOCl film—including peak attenuation and broadening—indicate successful encapsulation of the inorganic crystallites within the PVDF matrix, preserving the functional properties of both components.

**Figure 6:**
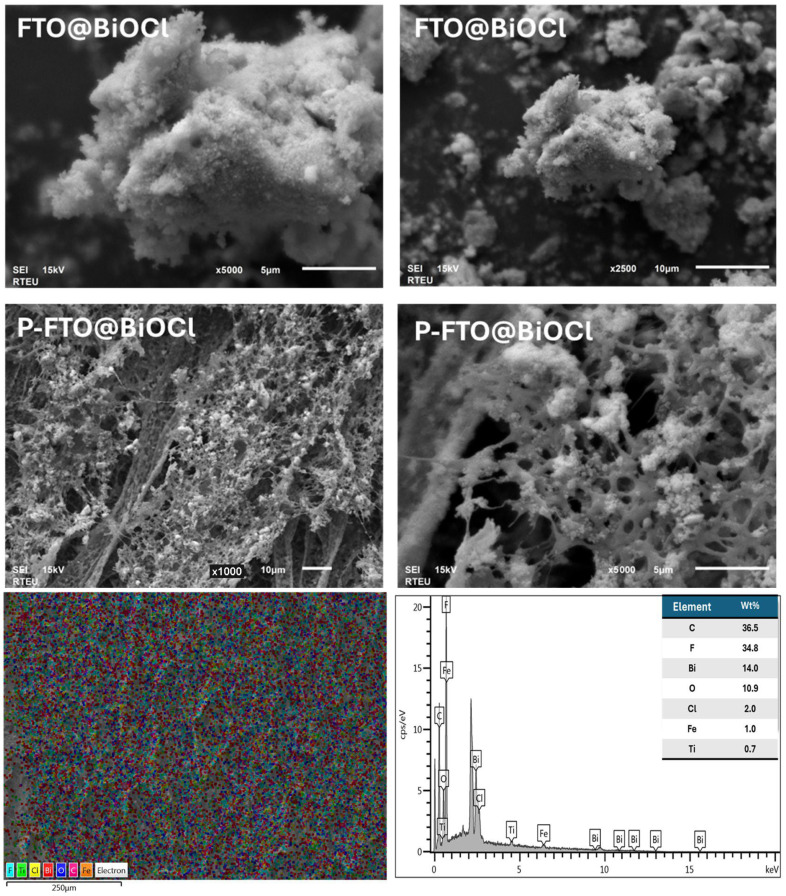
SEM images of FTO@BiOCl(III) and P-FTO@BiOCl(III) samples, elemental mapping, and EDS spectrum for P-FTO@BiOCl(III) sample.

The surface morphological features of the as-prepared catalysts were investigated using SEM. As observed in Figure 6, the FTO@BiOCl(III) composite exhibited an irregular and agglomerated particle morphology. The densely packed structure could be due to the placement of irregular spherical FeTiO_3_ particles between the cauliflower-like clusters of the BiOCl structure [48,51]. It was also clearly observed that upon introducing the FTO@BiOCl(III) composite heterostructure into the PVDF film, the irregularly shaped composite samples were distributed throughout the three-dimensional interconnected porous architecture of the polymeric thin film. The SEM images, therefore, revealed the successful assembly of P-FTO@BiOCl samples. Additionally, the relevant elemental mapping analysis and EDS spectrum (Figure 6) demonstrated the uniform distribution of Fe, Ti, O, Bi, Cl, C, and F across the P-FTO@BiOCl composite, further supporting the successful homogeneity of FTO@BiOCl(III) particles within the polymer matrix.

**Figure 7:**
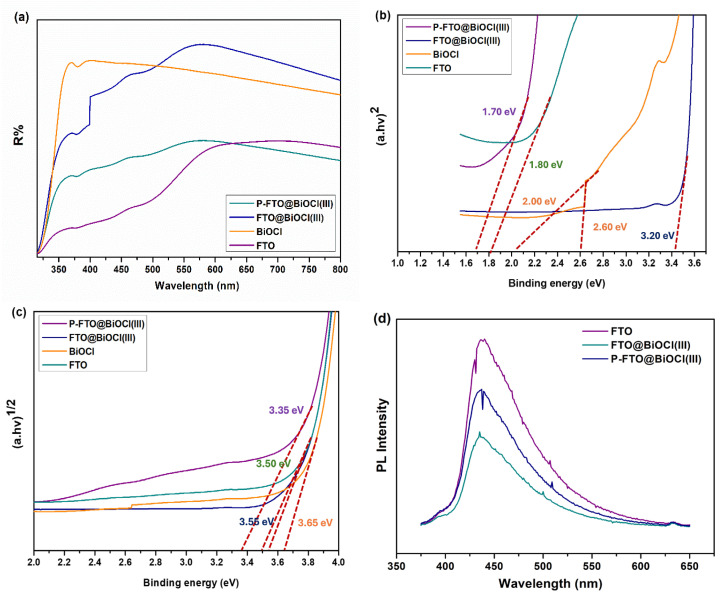
(**a**) UV–Vis DRS spectra, (**b**,**c**) Tauc plots, and (**d**) PL spectra of as-fabricated samples.

The light-harvesting abilities of the as-fabricated FTO, BiOCl, FTO@BiOCl, and P-FTO@BiOCl(III) samples were investigated using UV/Vis-DRS analysis. As clearly observed in Figure 7a, all synthesized samples exhibited sufficient light absorption ability across the entire UV-visible wavelength range. In the case of specific evolution, the sole FeTiO_3_ showed a gradual increase in the absorption profile from the UV edge (~350 nm) to the visible range (800 nm), with the highest light-absorbing behavior observed around 550–700 nm in the visible spectrum. That is, the FTO sample could exhibit visible light-driven activity across a broad wavelength range, arising from Fe^2+^ 3d–3d crystal-field transitions and O 2p → Fe 3d charge-transfer transitions [52]. Furthermore, the pristine BiOCl exhibited a sharp, intense absorption peak in the 370–380 nm range. This could be due to the intrinsic band-to-band electronic transition occurring from the hybridized Bi 6s–O 2p valence band structure, with a band gap ~3.2–3.5 eV [53]. The superior capability did not change markedly across the entire visible range from 400 to 800 nm. The extended efficient visible-light response could be ascribed to oxygen-vacancy-induced defect states within the band gap, leading to the formation of a sub-band-gap [54,55]. After integrating both catalysts, a notable enhancement in the overall absorption intensity was observed, particularly in the visible light region, due to the synergistic combination of their well-defined optical properties. This supported the successful formation of an electronically coupled FTO@BiOCl heterojunction with improved optical response. In addition, the broad and continuous absorption profile of P-FTO@BiOCl(III) suggested the potential of PVDF-based FTO@BiOCl(III) thin films for solar-driven photocatalytic applications. However, the overall absorbance intensity across the 350–800 nm range declined. The decrement could result from partial light-scattering effects introduced by the porous, semi-transparent PVDF film structure [56].

The optical band gap energies of the as-prepared samples were estimated as both direct (*n* = 2) and indirect (*n* = 1/2) transition values, using the Kubelka–Munk theory to explain their electronic structures (Figure 7b,c). The indirect band gap values were found to be in the range of 3.35–3.65 eV, while the direct band gap values were determined in relatively lower energies, suggesting the presence of interfacial electronic states and defects. As observed in Figure 7b, the FTO sample showed 1.70 eV of the direct band gap energy, whereas BiOCl exhibited two direct band gap energies (2.00 eV and 2.60 eV). The outcomes indicated that FTO could be explained by its stronger photoresponse as a semiconductor, and BiOCl had higher surface-adsorbed oxygen vacancies. After the formation of the FTO@BiOCl(III) heterostructure, the band gap value was shifted to 3.20 eV, which was higher than that of the pristine FTO and BiOCl samples. The increase in band gap values could be attributed to two main syngenetic effects: Firstly, the good distribution of BiOCl particles within the FeTiO_3_ structure, which altered the local electronic environment and redistributed electron–hole species at the heterojunction interface [44,57]. The electronic structure modified the band structure, resulting in an increase in the optical transition energy [57]. Secondly, the unique layered electronic structure of BiOCl introduced additional Bi-derived mid-band energy levels within the FeTiO_3_-based heterostructure [57,58]. The mid-band states partitioned the density of states near the band edges into discrete sub-levels, which contributed to the increase in the as-measured direct band-gap energy [44,58]. For P-FTO@BiOCl(III), the relevant band gap declined to 1.70 eV, suggesting that the PVDF matrix might arrange the electronic structure of the active heterojunction system via interfacial polarization effects and surface dipole interactions [56]. On the other hand, the indirect band gap energies were determined as 3.50 eV, 3.65 eV, 3.55 eV, and 3.35 eV for FTO, BiOCl, FTO@BiOCl(III), and P-FTO@BiOCl(III), respectively. The relatively high direct band gap energies are associated with enhanced oxidative potential, enabling efficient oxidation of organic compounds and the generation of reactive oxygen species (ROS) [59]. In addition, the PL analysis was performed to determine the extent of charge recombination in the as-obtained photocatalysts. As depicted in Figure 7d, the bare FTO sample exhibited the strongest PL emission peak, whereas after integrating BiOCl, the PL peak intensity decreased. It was suggested that the formation of the FTO@BiOCl heterostructure effectively suppressed charge carrier recombination compared to the pristine FTO. Notably, P-FTO@BiOCl(III) displayed a slightly higher PL intensity relative to the FTO@BiOCl(III) heterostructure, which was consistent with the observed photocatalytic performance trends. This slight increase might be attributed to the partial restriction of interfacial charge transfer pathways upon dispersion of the active heterojunction within the PVDF polymer matrix, thereby reducing the overall photocatalytic activity of the immobilized film system.

### 3.2. Photocatalytic Activities in Batch System

#### 3.2.1. Primarily Photocatalytic Studies

**Figure 8:**
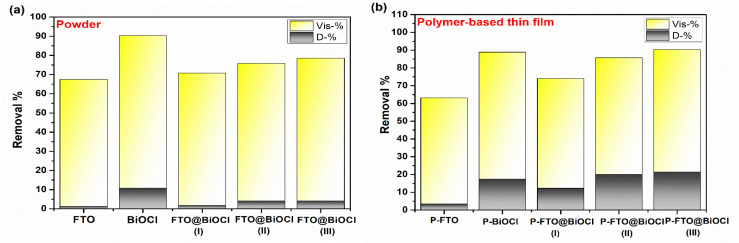
Photocatalytic removal efficiencies of (**a**) powder and (**b**) PVDF-included samples.

Photocatalytic antibiotic decomposition was systematically examined under visible light illumination in the presence of FTO, BiOCl, and their heterostructures, both in powder form and immobilized as PVDF-based thin films. As presented in Figure 8a, the pristine FeTiO_3_ showed 66.3% doxycycline (DOX) decomposition after 180 min of visible light irradiation. The integration of FTO with BiOCl improved antibiotic removal performance, with removal efficiencies calculated as 68.9%, 71.7%, and 74.4% for FTO@BiOCl(I), FTO@BiOCl(II), and FTO@BiOCl(III), respectively. The enhancement effect of BiOCl was due to the fact that: (i) BiOCl increased surface oxygen vacancies and defects on the surface, broadening the photo-assisted degradation rate; (ii) its layered tetragonal crystal structure promoted mass transfer; and (iii) its addition provided a strong electric field, increasing the separation rate of photo-induced electrons and holes. In addition to the above, BiOCl alone showed approximately 10% DOX adsorption efficiency owing to its functional groups, which also contributed to the adsorption performance of the heterostructure samples proportional to their BiOCl content (Figure 8a). Overall, it could be concluded that the improved DOX removal efficiency achieved through the formation of the FTO@BiOCl heterostructure and the transition from FTO@BiOCl(I) to FTO@BiOCl(III) clearly demonstrated that the higher BiOCl content in the composite enhanced the balance between light absorption, active site availability, and charge carrier dynamics, resulting in superior photocatalytic antibiotic degradation performance. In addition, a similar trend was also observed for the PVDF-including film systems: the decomposition efficiency was demonstrated as 59.7%, 71.4%, 61.4%, 65.7% and 68.9% for P-FTO, P-BiOCl, P-FTO@BiOCl(I), P-FTO@BiOCl(II) and P-FTO@BiOCl(III), respectively (Figure 8b). The photocatalytic performances of the PVDF-based thin films were found in lower values as compared to the corresponding powder catalysts. This reduction could be attributed to the presence of the PVDF matrix, which might limit light penetration and reduce the photon transfer rate to the active sites. Furthermore, the polymer layer could partially block accessible surface areas, reducing the number of available active sites. Specifically, the PVDF integration enhanced the dark adsorption. This could be attributed to the PVDF structure, which increases surface hydrophilicity and surface functionality, thereby facilitating stronger interactions with pollutant molecules. It was noteworthy that the P-FTO@BiOCl(III) catalyst showed a satisfactory photocatalytic ability toward visible light-driven antibiotic degradation, and thus, it was performed in further studies, herein.

In addition, the DOX degradation efficiency under visible light in the presence of a P-FTO@BiOCl(III) catalytic system was compared with those of other PVDF-based photocatalysts reported in the literature, and the results are listed in Table S1 [60,61,62]. Considering the initial antibiotic concentration, the P-FTO@BiOCl(III) system could be presented as a competitive and highly feasible approach for DOX removal.

#### 3.2.2. Effect of pH

**Figure 9:**
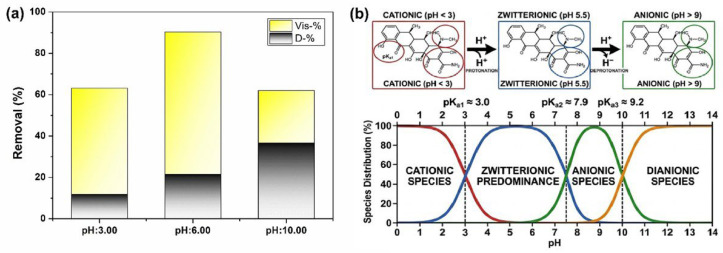
(**a**) pH effects on DOX degradation and (**b**) species distribution of DOX antibiotics.

In photocatalytic systems, the solution pH is considered a critical operating parameter that directly determines process efficiency, as it simultaneously affects both the catalyst surface charge and the ionization equilibrium of the target contaminant. DOX photodegradation experiments conducted using a P-FTO@BiOCl(III) film catalyst under pH 3.0, 6.0 and 10.0 conditions yielded total removal efficiencies of 63.1%, 90.3% and 62.1%, respectively; these differences can be directly attributed to the pH-dependent species distribution of DOX (Figure 9a). DOX antibiotic is an amphoteric tetracycline derivative with pK_a1_ = 3.0, pK_a2_ = 7.9 and pK_a3_ = 9.2 (Figure 9b). It exists as a cationic form (DOX^+^) at pH < 3, as a zwitterionic form in the pH 4–9 range, and as an anionic form (DOX^−^) at pH > 9 [63]. The highest total removal efficiency was achieved at pH 6.0 (Vis = 68.9%, Dark = 21.4%). Under these conditions, DOX predominantly exists in a zwitterionic form; this is the most favorable form for strong interaction with the catalyst surface via both π–π stacking interactions and Lewis acid–base coordination. At the same time, •OH radical generation occurs efficiently at pH 6.0, and the radical scavenging pressure associated with the high H^+^ concentration remains at a negligible level. This finding is consistent with the literature, which reports that pH 6.0 provides the optimal condition for DOX degradation in similar heterojunction systems [64]. At pH 3.0, the visible degradation value has decreased to 51.3%. Under these conditions, as DOX is at the pK_a1_ = 3.0 threshold, it largely converts to its cationic form; the electrostatic interactions governing access to the molecule’s reactive surface regions create a negative equilibrium. Furthermore, the high H^+^ concentration directly competes with •OH radicals [65], thereby limiting the effectiveness of ROS against DOX. It should not be overlooked that the presence of the PVDF matrix may also limit the catalyst’s contact surface with DOX under acidic conditions, as surface-dependent degradation mechanisms in film systems are more sensitive to disruptions in surface charge balance compared to powdered catalysts. Nevertheless, the achievement of a visible light contribution of 51.3% even at pH 3.0 demonstrates that the FTO@BiOCl heterojunction retains a certain level of photocatalytic activity via direct hole (h^+^) oxidation and superoxide radical (•O_2_^−^) mechanisms, despite these unfavorable conditions. At pH 10.0, an interesting picture emerges: whilst the highest dark adsorption (36.6%) is obtained under these conditions, it is also at this pH that photodegradation drops to its lowest value (25.4%). The high dark adsorption value can be explained by the ability of DOX’s enol and carbonyl groups to form strong coordination complexes with Bi^3+^ Lewis acid centers in a basic environment, despite the electrostatic repulsion between the anionic DOX and the negatively charged catalyst surface [66]. The fact that P-FTO@BiOCl(III) achieves the highest total DOX removal (90.3%) at a near-neutral pH of 6.0 indicates that the system operates most effectively in the presence of zwitterionic DOX, where dark adsorption and visible-light-induced photodegradation are synergistically optimized.

#### 3.2.3. Effect of Scavengers and Photocatalytic Degradation Mechanism

**Figure 10:**
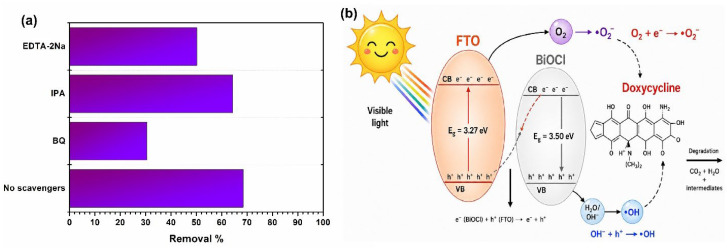
Effect of scavengers (**a**) and the as-presented possible heterojunction mechanism (**b**).

To understand the decomposition mechanism, trapping-agent experiments were carried out using IPA, BQ and EDTA-2Na, as trapping agents for hydroxyl radicals (•OH), superoxide radicals (•O_2_^−^), and photo-indued holes (h^+^), respectively. As shown in Figure 10, the addition of BQ significantly hindered the catalytic activity of P-FTO@BiOCl(III) (30.5%), suggesting that the superoxide radicals were the most dominant species for the DOX reduction. Furthermore, the addition of EDTA-2Na addition slightly decreased the activity (50.2%), indicating that the photogenerated holes contributed to the degradation process. Additionally, IPA exhibited no effect on the reaction (64.4%), stating that the hydroxyl radicals (•OH) did not play a role in the removal system.

On the other side, the indirect band structures derived from the Tauc plot and the related band alignments of FTO and BiOCl powders were analyzed in detail to elucidate possible heterojunction mechanisms of the as-fabricated P-FTO@BiOCl(III) sample. The band gap values of FTO and BiOCl were determined as 3.50 eV and 3.65 eV, respectively, in this study. Moreover, the relevant valence band (VB) and conduction band (CB) potentials of the photocatalysts were calculated by using the following equations (Equations (2) and (3)):

E_CB_ = *χ* − E_e_ − 0.5 E_G_ (2)

E_G_ = E_VB_ − E_CB_ (3)

where E_e_, E_G_ and χ were attributed to the energy of the free electrons on the hydrogen scale (4.50 eV), the band gap energies and the absolute electronegativity, respectively. The electronegativities were determined as 5.70 eV and 6.64 eV for FTO and BiOCl, respectively. [67]. Thus, for FTO, the E_CB_ and the E_VB_ potentials were determined as −0.55 eV and 2.95 eV, respectively, and for BiOCl, the E_CB_ and the E_VB_ potentials were calculated as 0.32 eV and 3.97 eV, respectively. According to these, the CB value of FTO exhibited more highly negative potential than that of the O_2_/•O_2_^−^ redox potential (−0.35 eV vs. NHE), resulting in a strong reduction feature. The VB value of BiOCl was found to be more positive than the standard redox potentials (OH/H_2_O = +2.8 eV vs. NHE and OH/OH^−^ = +2.38 eV vs. NHE), confirming its excellent oxidation properties. It was therefore stated that before contact, FTO and BiOCl behaved as a reduction photocatalyst (RP) and oxidation photocatalyst (OP), respectively. After contact, the electron transfer occurred from BiOCl (RP) to FTO (OP) until the thermodynamic equilibrium was achieved at the interfaces. Then, under visible light irradiation, the photoinduced electrons on the CB of BiOCl transferred to the VB of FTO, and the highly reactive photogenerated electrons and holes were retained in the CB of FTO and VB of BiOCl. This allowed electrons in the CB of FTO and holes in the VB of BiOCl with strong reduction and oxidation abilities, respectively, to persevere. When the results of BQ-and IPA-included experiments were evaluated, it could be indicated that the strong electrons in the CB of FTO participated in the decomposition by generating the superoxide radicals, whereas the active holes in the VB of BiOCl directly degraded DOX molecules. The possible reaction mechanism was proposed in Figure 10b and the relevant reactions could be presented as follows:

P-FTO@BiOCl(III) + hv → e^−^(FTO) + h^+^(BiOCl) + e^−^(FTO) + h^+^(BiOCl) (4)

e^−^ (FTO)_CB_ + O_2_ → •O_2_^-^ (5)

(•O_2_^−^, h^+^(BiOCl)_VB_) + DOX → CO_2_ + H_2_O + intermediate (6)

### 3.3. Photocatalytic Activities in Air-Lift Reactor System

**Figure 11:**
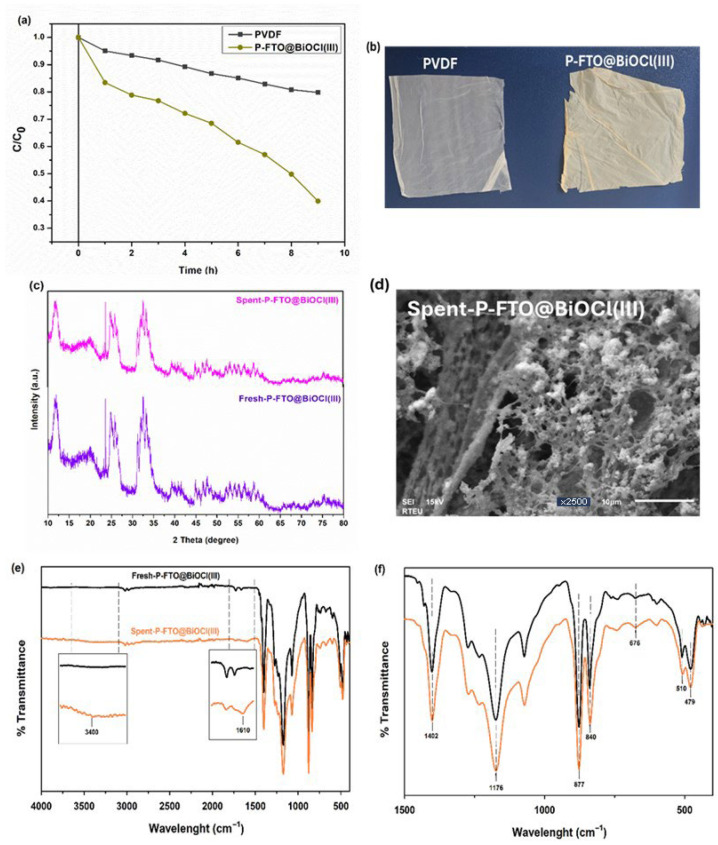
(**a**) Photocatalytic activity in airlift reactor, (**b**) photographs of as-fabricated films, (**c**) XRD patters of fresh and spent-P-FTO@BiOCl(III), (**d**) SEM image of spent-P-FTO@BiOCl(III) and (**e**) FTIR spectra of fresh and spent-P-FTO@BiOCl(III) (inset) with magnified plots and (**f**) enlarged FTIR spectra of fresh and spent-P-FTO@BiOCl(III).

The photocatalytic performance of the as-prepared PVDF and the optimal P-FTO@BiOCl(III) film was evaluated in an air-lift reactor under visible light irradiation. As shown in Figure 11a, the sole PVDF thin film demonstrated negligible degradation activity, with only approximately 20% removal performance after 9 h, confirming that the polymer matrix itself did not contribute to the photocatalytic degradation process. After the addition of FTO@BiOCl(III) into PVDF, the degradation efficiency significantly improved, achieving approximately 65% removal within the same time period. The removal behavior exhibited a continuous, gradual decrease, indicating that the catalytic film maintained stable and sustained photocatalytic activity throughout the reaction time. The result suggested that the air-lift reactor configuration provided adequate mixing and high contact area, ensuring sufficient oxygen availability and light penetration to establish desirable catalytic performance for large-scale P-FTO@BiOCl(III) film, which could be observed in Figure 11b. Furthermore, the spent-P-FTO@BiOCl(III) film was characterized by XRD, SEM, and FTIR methods to examine the applicability of the film. As depicted in the XRD spectra (Figure 11c), after the long-term reaction, all characteristic peaks of the individual components were preserved without the appearance of new peaks or the disappearance of existing ones, indicating structural stability. However, a slight decrease in peak intensities was observed, which could be attributed to surface coverage by reaction intermediates and to partial structural disorder induced during the photocatalytic process. Moreover, the reaction did not show any effect on the morphological structure, observing no detectable changes in the SEM image of the spent-P-FTO@BiOCl(III) film (Figure 11d). In addition, the as-spent (P-FTO@BiOCl(III)) was analyzed by FTIR spectroscopy. Clearly, the characteristic bands of BiOCl and FeTiO_3_ remained unaltered, confirming that neither the inorganic filler nor the polymer backbone underwent chemical degradation during the adsorption process (Figure 11e). Similarly, as shown in Figure 11f as an enlarged FTIR spectrum, importantly, no significant shifts or disappearance of the principal P-FTO@BiOCl(III) absorption bands were detected upon DOX loading, indicating that the structural integrity of the polymer matrix was fully preserved. Furthermore, two notable spectral changes were observed in the spent-P-FTO@BiOCl(III) film. First, the broad absorption band in the 3300–3500 cm^−1^ region, attributable to overlapping O–H and N–H stretching vibrations, became markedly more intense compared to the fresh-P-FTO@BiOCl(III) films prior to DOX exposure. This enhancement was consistent with the presence of the hydroxyl and amino functional groups characteristic of the DOX molecule on the film surface. Second, a new absorption band emerged at approximately 1610 cm^−1^, which was assigned to the C=O stretching vibration of the chelated/conjugated carbonyl group of DOX, a region where fresh-P-FTO@BiOCl(III) film exhibited no characteristic absorption [20]. According to this, the chemical structure of the P-FTO@BiOCl(III) film remained unchanged during the antibiotic decomposition, and the relevant functional groups were able to adsorb DOX molecules. Overall, the P-FTO@BiOCl(III) thin film could be considered as a promising candidate for efficient visible-light-driven DOX degradation in an air-lift reactor with significant structural stability.

## 4. Conclusions

In this study, the performance of photocatalytic thin-film systems developed by integrating FeTiO_3_/BiOCl heterojunction structures into a PVDF matrix was comprehensively evaluated. Characterization results demonstrated that the heterojunction structure was successfully formed and that a homogeneous distribution was achieved within the polymer matrix. UV-DRS analyses revealed that light absorption increased and charge carrier separation improved upon heterostructure formation. In photocatalytic results, pure FeTiO_3_ achieved a removal efficiency of 66.3% within 180 min, whereas the FTO@BiOCl(III) heterojunction demonstrated the highest performance, reaching 74.4%. In PVDF-based thin-film systems, the best example, P-FTO@BiOCl(III), provided a removal efficiency of 68.9%. In pH studies, optimal performance was achieved at pH 6.0, with a total removal of 90.3%. Radical trapping experiments demonstrated that superoxide radicals play a dominant role and that photogenerated radicals contribute to the degradation process. In experiments conducted in the airlift reactor system, the P-FTO@BiOCl(III) film demonstrated stable performance, achieving approximately 65% removal after 9 h. Post-reaction XRD, SEM, and FTIR analyses confirmed that the catalyst retained its structural integrity. These results underline the ability of the PVDF-supported FeTiO_3_/BiOCl heterojunction thin film to maintain structural integrity and sustained activity under continuous conditions, offering a promising pathway toward practical photocatalytic water treatment technologies.

## Data Availability

The raw data supporting the conclusions of this article will be made available by the authors on request.

## Supplementary Materials

The following supporting information can be downloaded at: https://www.mdpi.com/article/10.3390/polym18101246/s1.
