# Supplementary material for: Heterojunction FeTiO3/BiOCl Photocatalytic Polymer Film in an Airlift Reactor: Efficient Visible-Light Degradation of Pharmaceutical Pollutant
Source: Polymers (Basel). 2026 May 20;18(10):1246. doi: 10.3390/polym18101246 (PMC13210920; doi:10.3390/polym18101246)
Supplement: Supplementary file 1 [file polymers-18-01246-s001.zip › polymers-4318705-supplementary.pdf]

Supplementary Information

Heterojunction FeTiO₃/BiOCl photocatalytic polymer film in an airlift reactor: Efficient visible-light degradation of pharmaceutical pollutant

Nergiz Kanmaz[1], Nese Cakir Yigit [2],*and Özlem Tuna[1],*

[1] Department of Chemical Engineering, Faculty of Engineering, Yalova University, 77200, Yalova, Turkey
[2] Department of Polymer Materials Engineering, Faculty of Engineering, Yalova University, 77200, Yalova, Turkey
* Correspondence: nese.cakir@yalova.edu.tr (Nese), ozlem.tuna@yalova.edu.tr (Özlem)

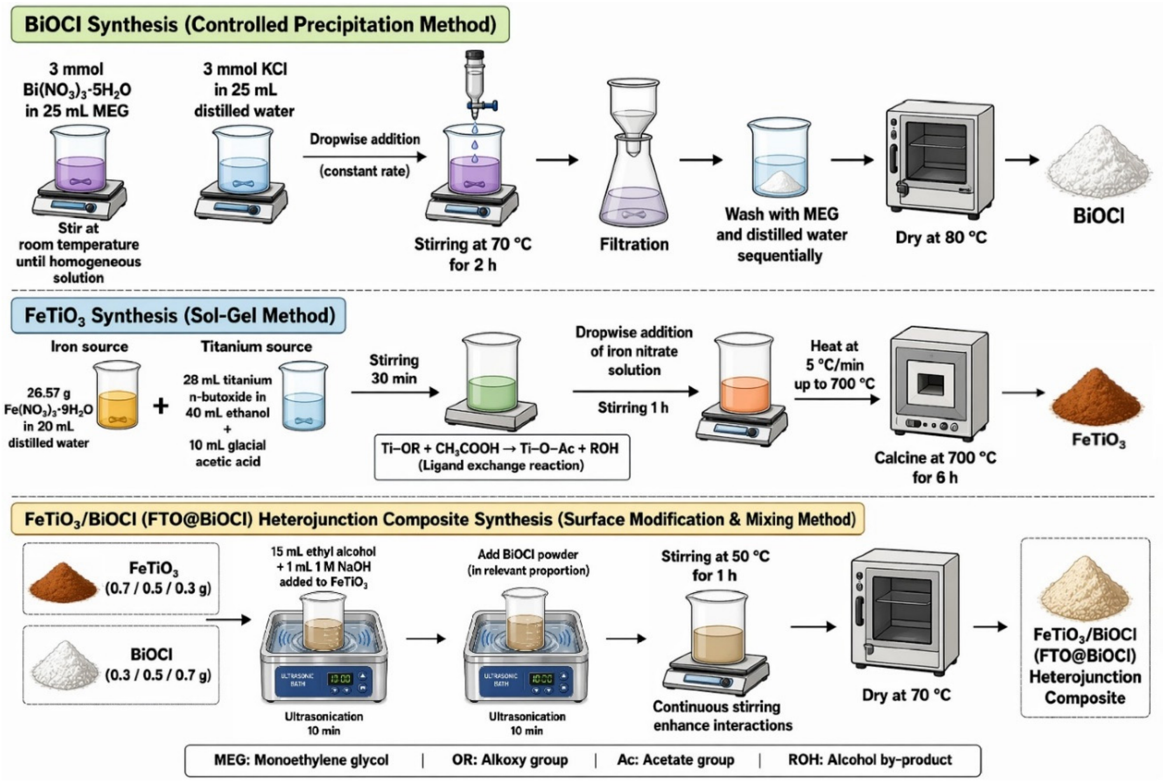

Figure S1. Schematic representation of BiOCl, $FeTiO_3$, and $FeTiO_3$@BiOCl heterostructures

Table S1: Comparison with other PVDF-based thin film photocatalysts for the degradation of doxycycline

Film Sample	Degradation Efficiency	Initial DOX Concentration (ppm)	Time (min)	Ref
$BiVO_4$/g–C_3N_4/PVDF	94.9	5	240	[1]
Lignin-coated PVDF	85.5	-	120	[2]
TiO_2/PVDF	85.9	-	60	[3]
**P-FTO@BiOCl(III)**	**68.9**	**10**	**120**	**Current Study**

[1] Wang, Q., Dong, Y., Han, J., Zhu, J., Li, Z., Zhang, F., ... & Wang, P. (2025). Polydopamine introduced chemical bonds to generate a $BiVO_4$/g–C_3N_4–functionalized PVDF photocatalytic membrane with high stability for continuous dynamic removal of doxycycline. *Journal of Membrane Science*, *733*, 124345.

[2] Xu, Y., Leo, C. P., & Hu, Z. (2025). Photoelectrocatalytic regeneration of adsorptive lignin-coated PVDF membranes for antibiotic removal during microfiltration. *Journal of Water Process Engineering*, *76*, 108104.

[3] Thao, T. Q., Tien, N. T. C., Vy, V. C. T., Thuy, N. T., Thi, P. T., Lei, J., & Huy, N. N. (2025). Insights into the degradation of doxycycline in water using electrochemically prepared TiO_2: Effect of environmental factors, reaction mechanism, degradation pathway, and toxicity evaluation. *Journal of King Saud University–Science*, *37*.
